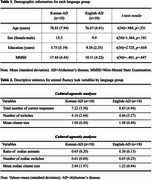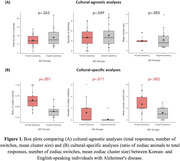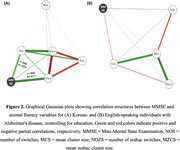# Cultural‐linguistic divergences in animal fluency performance between Korean‐ and English‐speaking older adults with Alzheimer's disease

**DOI:** 10.1002/alz70858_101960

**Published:** 2025-12-25

**Authors:** Junyoung Shin, Adolfo M. Garcia, Michael Scimeca, Swathi Kiran, Jee Eun Sung

**Affiliations:** ^1^ Ewha Womans University, Seodaemun‐gu, Seoul, Korea, Republic of (South); ^2^ Universidad Santiago de Chile, Estación Central, Santiago de Chile, Chile; ^3^ Global Brain Health Institute, University of California, San Francisco, CA, USA; ^4^ Cognitive Neuroscience Center (CNC), Universidad de San Andres, Buenos Aires, CA, Argentina; ^5^ Cognitive Neuroscience Centre, University of San Andres, Victoria, Buenos Aires, Argentina; ^6^ Boston University, Boston, MA, USA

## Abstract

**Background:**

Verbal fluency tasks reveal the influence of cultural‐linguistic backgrounds on word retrieval. A recent cross‐linguistic study on animal fluency in Korean‐ and English‐speaking individuals with stroke aphasia found that Korean‐speakers generated more zodiac animals rooted in East Asian‐culture than English‐speakers. Their findings highlight the importance of considering cultural factors in word retrieval and the need for further research in other neurological communication disorders. We examine whether cultural differences in animal fluency emerge between Korean‐ and English‐speaking individuals with Alzheimer's disease (AD) by analyzing clustering and switching patterns across both traditional Western‐based and zodiac animal categories, assessing their potential as predictors of cognitive function.

**Method:**

Thirty‐six participants (18 Korean‐AD, 18 English‐AD) were matched for age, sex, and Mini‐Mental State Examination (all *p* > .05), except for education (*p* < .05). During a 60‐second animal fluency task, participants' responses were analyzed using culture‐agnostic analyses based on traditional criteria: (1) total number of correct responses, (2) number of switches (NOS), and (3) mean cluster size (MCS). Culture‐specific analyses followed previous work: (1) ratio of zodiac animals to total responses (zodiac ratio), (2) number of zodiac switches (NOZS), and (3) mean zodiac cluster size (MZCS). Group differences were examined using generalized linear mixed models, with stepwise regression identifying cognitive function predictors.

**Result:**

Groups showed comparable performance in culture‐agnostic analyses, with no significant differences in total correct responses, NOS, or MCS (all *p* > .05, Table 1). In culture‐specific analyses, Korean‐AD produced a significantly greater zodiac ratio (*p* < .001), NOZS (*p* = .011), and MZCS (*p* = .002) compared to English‐AD (Figure 1). The stepwise regression model identified NOZS as the strongest predictor of MMSE for the Korean‐AD (*p* = .009, *R* ^2^ = .354), while no variables predicted MMSE for the English‐AD (Figure 2).

**Conclusion:**

These findings demonstrate that animal fluency tasks may elicit culturally‐distinct retrieval patterns in AD, consistent with previous findings in stroke aphasia. Culture‐specific categories reflecting East Asian features were more strongly linked to cognitive decline in Korean‐AD, suggesting that culturally‐sensitive analyses of verbal fluency offer valuable clinical insights.